# Supplementary material for: Development of a recombinant single-cycle influenza viral vector as an intranasal vaccine against SARS-CoV-2
Source: Sci Rep. 2026 Jun 13;16:27047. doi: 10.1038/s41598-026-55581-y (PMC13522583; doi:10.1038/s41598-026-55581-y)
Supplement: Supplementary file 1 [file 41598_2026_55581_MOESM1_ESM.pdf]

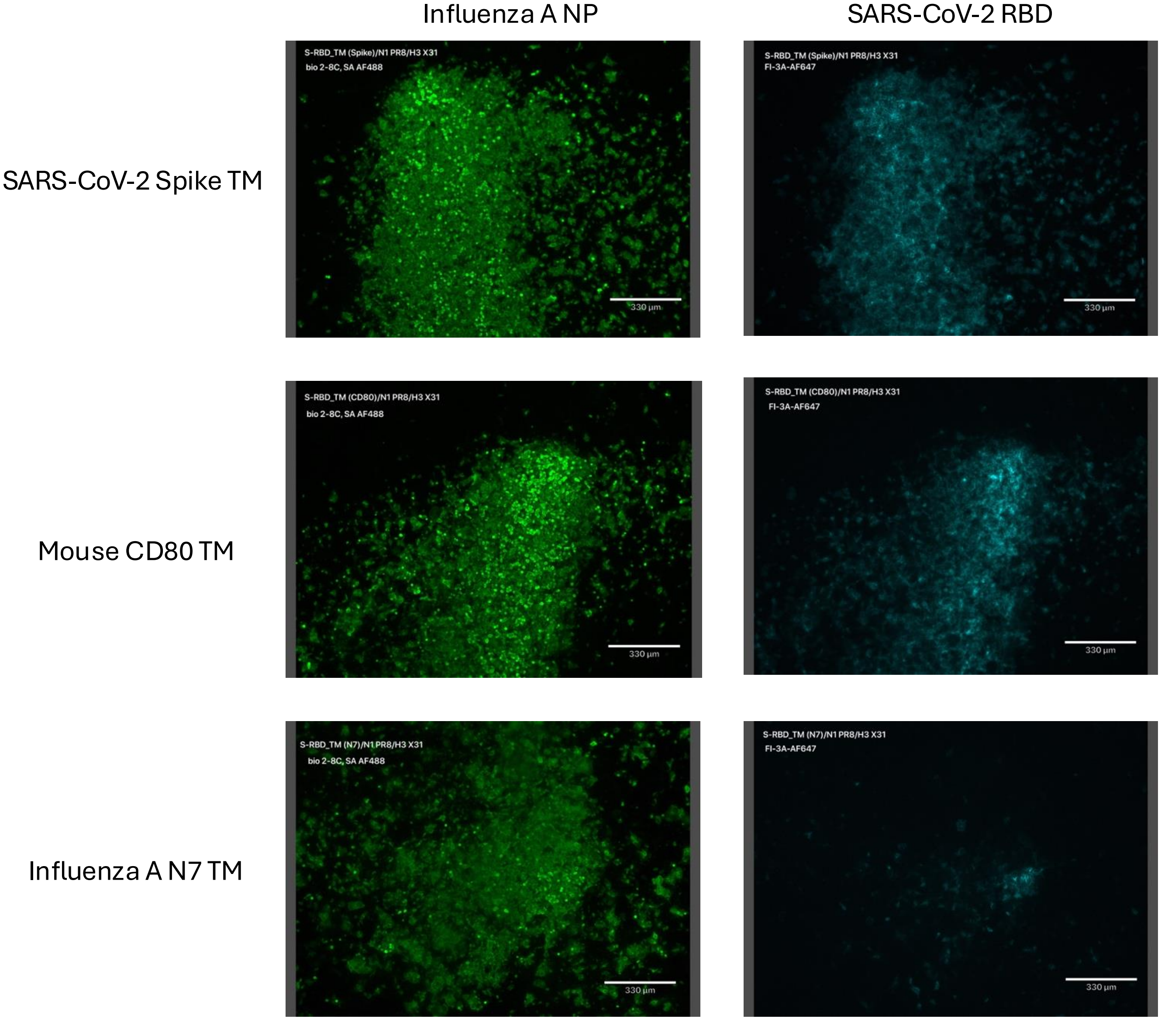

Figure S1: Expression of influenza NP and RBD in cell culture infected with S-RBD-TM with various TM. MDCK cells seeded in 6-well plate were infected with S-RBD-TM (fused to a SARS-CoV-2 Spike TM, mouse CD80 TM or an influenza neuraminidase N7 transmembrane domain). 16 hours post infection, cells were formalin fixed, permeabilised with 0.5% Triton-X 100 and stained with an RBD-specific IgG1 (FI-3A) labelled with AF647, and a biotinylated anti-Influenza NP IgG1 (2-8C biotin), followed by Streptavidin labelled with AF488. Stained cells were visualised using a fluorescence microscope. Scale bar: 330uM. (TM: transmembrane domain, AF647: Alexa Fluor 647, AF488: Alexa Fluor 488)

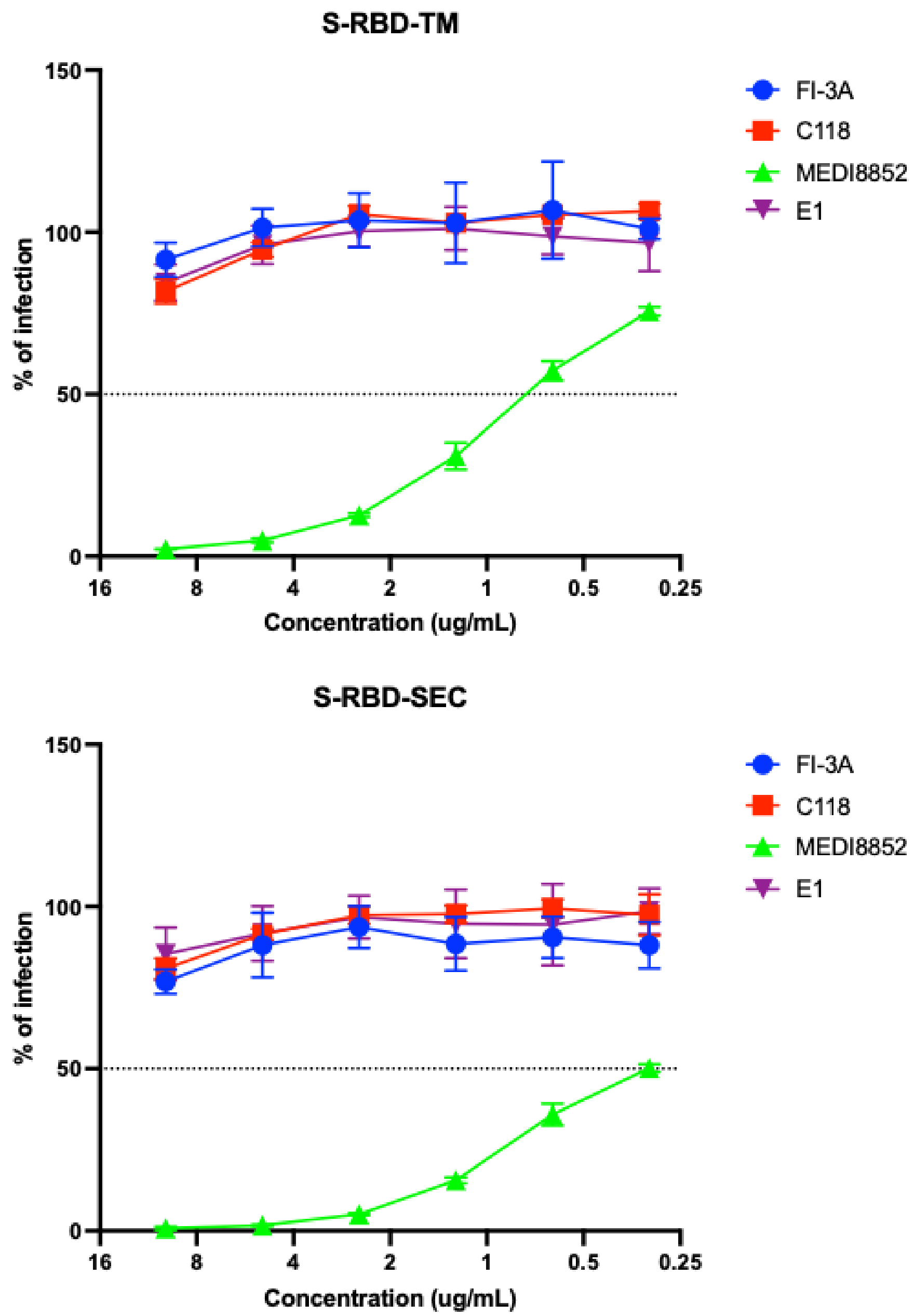

Figure S2: Neutralisation of S-RBD-TM and S-RBD-Sec viruses by anti-RBD and anti-HA monoclonal antibodies. S-RBD-TM and S-RBD-Sec viruses were incubated with serial dilutions of anti-RBD neutralising monoclonal antibodies (FI-3A and C118), anti-HA neutralising antibody (MEDI8852) or an irrelevant antibody (E1) prior to infection of target cells (MDCK-SIAT1). Infection was quantified relative to no-antibody controls and expressed as percentage infection. Data represet mean±SEM from 2 technical replicates.

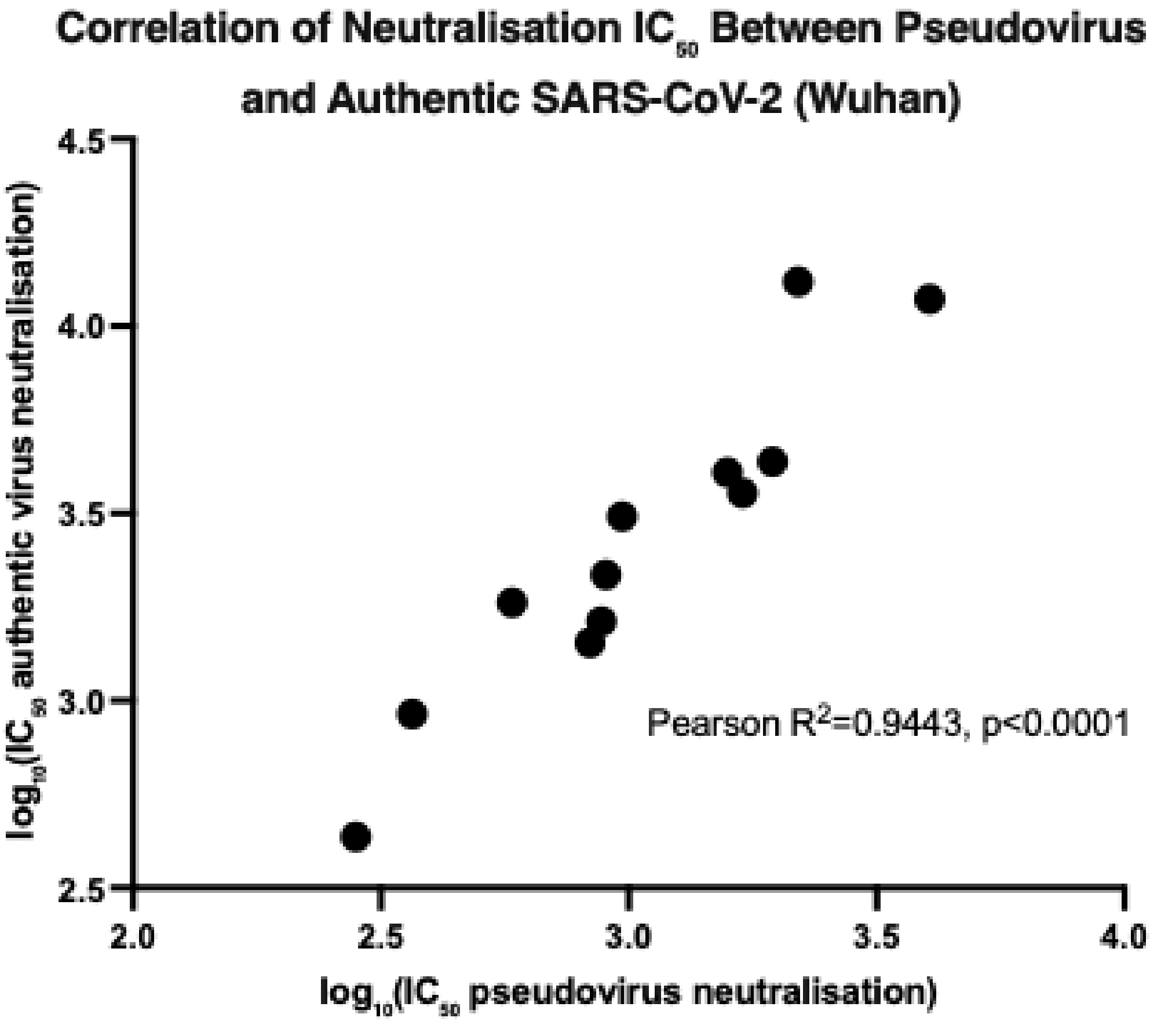

Figure S3: Correlation of neutralisation between SARS-CoV-2 Wuhan authentic virus neutralisation (SARS-CoV-2 Wuhan) and pseudovirus neutralisation assays.

SARS-CoV-2 Variant	G339	R346	S371	S373	S375	K417	N440	G446	L452	N460	S477	T478	E484	F486	Q493	G496	Q498	N501	Y505
Wuhan	.	.	.	.	.	.	.	.	.	.	.	.	.	.	.	.	.	.	.
Alpha (B.1.1.7)	.	.	.	.	.	.	.	.	.	.	.	.	.	.	.	.	.	N501Y	.
Beta (B.1.351)	.	.	.	.	.	K417N	.	.	.	.	.	.	E484K	.	.	.	.	N501Y	.
Gamma (P.1)	.	.	.	.	.	K417T	.	.	.	.	.	.	E484K	.	.	.	.	N501Y	.
Delta (B.1.617.2)	.	.	.	.	.	.	.	.	L452R	.	.	T478K	.	.	.	.	.	.	.
Omicron BA.1	G339D	.	S371L	S373P	S375F	K417N	N440K	G446S	.	.	S477N	T478K	E484A	.	Q493R	G496S	Q498R	N501Y	Y505H
Omicron BA.2	G339D	.	.	.	.	K417N	N440K	.	.	.	S477N	T478K	E484A	.	Q493R	.	Q498R	N501Y	.
Omicron BA.4 / BA.5	G339D	.	.	.	.	K417N	N440K	.	L452R	.	S477N	T478K	E484A	F486V	Q493R	.	Q498R	N501Y	.
BA.2.75	G339D	.	.	.	.	.	N440K	G446S	.	.	S477N	T478K	E484A	.	Q493R	.	Q498R	N501Y	.
XBB / XBB.1.5	G339D	R346T	.	.	.	.	.	G446S	.	N460K	.	.	.	.	.	.	.	.	F486P / F486S

Table S1: Position-by-position mutation matrix of the SARS-CoV-2 spike RBD (residues 319–541). The table shows the specific amino acid changes in major variants relative to Wuhan SARS-CoV-2. Each column represents an RBD residue and each row a variant. Cells list the observed amino acid substitution at each position; blank cells indicate identity with the reference sequence.
